# Young people’s awareness on biobanking and DNA profiling: results of a questionnaire administered to Italian university students

**DOI:** 10.1186/s40504-017-0055-9

**Published:** 2017-06-10

**Authors:** Pamela Tozzo, Antonio Fassina, Luciana Caenazzo

**Affiliations:** Department of Molecular Medicine, University of Padova, via Falloppio 50, 35121 Padova, Italy

**Keywords:** Biobank, DNA forensic databases, Public attitudes, Questionnaire

## Abstract

Current policy approaches to social and ethical issues surrounding biobanks manifest lack of public information given by researchers and government, despite the evidence that Italian citizens are well informed about technical and other public perspectives of biotechnologies. For this reason, the focus of our survey was to interview our University’s students on these aspects. The sample consisted of Padua University students (*N* = 959), who were administered a questionnaire comprising eight questions covering their knowledge about biobanks, their perception of the related benefits and risks, their willingness to donate samples to a biobank for research purposes, their attitude to having their own DNA profile included in a forensic DNA database, and the reasons behind their answers. The vast majority of the students invited to take part in the survey completed the questionnaire, and the number of participants sufficed to be considered representative of the target population. Despite the respondents’ unfamiliarity with the topics explored, suggested by the huge group of respondents answering “I don’t know” to the questions regarding Itaian regulation and reality, their answers demonstrate a general agreement to participate in a biobanking scheme for research purposes, as expressed by the 91% of respondents who were reportedly willing to donate their samples. As for the idea of a forensic DNA database, 35% of respondents said they would agree to having their profile included in such a database, even if they were not fully aware of the benefits and risks of such action.

This study shows that Italian people with a higher education take a generally positive attitude to the idea of donating biological samples. It contributes to empirical evidence of what Italy’s citizens understand about biobanking, and of their willingness to donate samples for research purposes, and also to have their genetic profiles included in a national forensic DNA database. Our findings may have clear implications for the policy discussion on biobanks in Italy, in particular it is important to take into account the Italian population’s poor consciousness of forensic DNA database, in order to ensure a better interaction between policy makers and citizens and to make them more aware of the need to balance the individual’s rights and the security of society.

## Introduction

Biobanks are storage facilities used to assemble, preserve, and manage collections of biological samples. While the collection of such samples and data for research purposes has a long history in our education and healthcare systems, the recent increase in the number, size, and importance of biobanks has drawn attention to the changing nature of biomedical research, and of the relationships between investigators, participants in research, and the organizations that fund and manage biobanking facilities.

Advances in DNA technology, and the discovery of DNA polymorphisms have facilitated the creation of databases of individuals’ DNA for use in investigating crime. This has opened up a considerable range of opportunities for criminal investigations: comparing the DNA profiles of biological evidence found at the scene of a crime with those in the database can lead to the identification of the likely perpetrator of the crime. Forensic DNA databases collect, store and use the DNA profiles of named suspects, convicted offenders, victims, volunteers, and other people of potential interest to the police, subject to specific national regulations. Logically and theoretically, the higher the number of citizens whose DNA has been profiled and recorded in a database, the better the chances of identifying a criminal and that DNA databse expansion is part, in some Countries, of national governments’ attempts to ensure that more offenders are prosecuted and that criminals are deterred from reiterate the offences upon release (Zadok et al. 2010, Machado and Prainsack 2012). However, Santos et al. have published that the comparison of person-stain match figures in the context evaluated in their article would indicate that the expansion of a forensic DNA database does not necessarily mean obtaining greater performance in terms of person-stain matches (Santos et al., 2013). Indeed, although many national DNA databases are large, they don’t contain DNA of all citizens. This means that even if DNA is retraived from a crime scene, unless the perpetrator’s DNA is already in the database, it won’t necessarily generate a match. Proponents of a wide-population forensic DNA database argue that it may put an end to ethnic biases in existing databases, saving police efforts and money in the long run, as well as minimize persecution of the population. However, the counter argument is that the expected benefit from population-wide forensic DNA database, on the other hand, will impact on the balance between privacy rights of the individual and society needs for security, leading to a “surveillance society” (Zadok et al. 2010). Furthermore, as explained by Machado and Prainsack (2012), the issue of forensic DNA database expansion is far from being “black-and-white”, because the situation is more complex than that: some of those supporting the expansion of DNA databases, or the contexts in which DNA data should be used in this context, are driven by the same values and concerns as some of those who oppose database expansion: justice, privacy an the prevention and the correction of miscarriage of justice.

Both biobanks and forensic DNA databases have the potential to offer great social benefits. Biobanks are seen as one of the most promising means for personalizing medical care and improving public health (Budimir et al. 2011; Hewitt 2011; Milani et al. 2015). Forensic DNA databases are considered as tools for improving efforts to detect crime and identify suspects, and the end result is expected to be a reduction in crime rates, and greater public safety and security (Van Camp and Dierickx 2007; Patyn and Dierickx 2010; Ge et al. 2014). Critics argue, however, that operating forensic DNA databases carries potential threats to the protection of a range of human rights, particularly as concerns our freedom, autonomy, privacy, moral and physical integrity, and the presumption of our innocence; and the expansion of DNA databases might be perceived by the general population as an excessive form of state control.

In recent years, the importance of biobanks has been confirmed by the huge increase in the body of scientific literature concerning novel applications, and new lines of research in both public and private contexts. Generally speaking, however, when it comes to proposals for new, particular biobanking models (prompted by the rapid evolution of this field due to its enormous potential), a problem still remains. In fact, most citizens have never even heard of biobanks. Many appear reluctant to be involved in biobank-related research, whether as donors of samples to biobanks or as participants in cohort studies. At the same time, some European citizens have voiced concern that biobank-related research might turn against them, either by violating their right to confidentiality or proving disadvantageous in terms of influencing their insurance premiums, or employment opportunities, or prompting other forms of discrimination.

The development of biobanking for the purposes of medical research relies heavily on the trust and goodwill of participants volunteering to provide samples and, usually, also personal details about their health and living habits. The idea of being involved in biobanks seems to be readily accepted by most citizens, whereas society’s perception of the reliability of the techniques and analyses used in forensic DNA databases, and how they are regulated, may be perceived as a form of excessive state control (Caenazzo and Dierickx 2012).

Public engagement in biobanking has generated a rising trend in the number of samples/data included, and in the related information flows. In promoting public engagement, it is crucially important to encourage members of society to see the value to the community and give priority to the common good vis-à-vis an individual’s own interests, and this means promoting the public’s trust (Meslin 2010; Caenazzo et al. 2013; Critchley et al. 2015).

A key requirement for community engagement in biobanks and forensic DNA databases is to ensure that consultation goes beyond providing information and reassuring participants. It should include a respectful, genuine debate and dialogue between institutions, governments, researchers and citizens (Tupasela 2015; De Vries et al. 2016).

Although there are numerous policy guidelines on how to achieve this engagement, biobanks adopt a variety of different strategies to engage the public, drawing on a broad range of influences and activities focusing on the ethical, legal and social aspects of biobanks and DNA databases. One important example of such engagement strategies has often consisted in the use of population surveys as a form of community consultation (Hemminki et al. 2009; Cadigan et al. 2013; Ahram et al. 2014; Boeckhout and Douglas 2015).

In Italy, little is known about the level of public awareness concerning biobanks and the National Forensic DNA Database, and their acceptance by the country’s citizens. Hence our interest in assessing a sample of Italian adults on their knowledge and acceptance of the concept of large-scale biobanking and DNA profiling, as done previously by other European groups of researchers (Van Camp and Dierickx 2007; Machado and Silva 2014; Zieger and Utz 2015).

The importance of knowing Italians’ perception on forensic DNA database derives from the fact that Italy ratified the Prȕm Treaty with its own Law No. 85 (of 30th June 2009), which governs the institution of DNA databases. Its 33 articles establish that the purpose of DNA databases is to enable (Art. 7): - the collection of DNA profiles of: individuals who commit a crime (as detailed in Art. 9); biological evidence found at crime scenes; missing persons and their relatives; unidentified bodies or parts thereof; and the comparison of DNA profiles for personal identification purposes.

The law calls for the institution of a central laboratory responsible for the analysis of samples to ascertain the DNA profiles of individuals and of biological evidence, and for the storage of biological samples from which said DNA profiles have been obtained (Art. 8).

Article 12 regulates the security measures against unauthorized access to the data. Article 13 concerns the destruction of the biological samples and genetic profiles of convicted persons, after a maximum storage period of 20 and 40 years, respectively, unless the convicted person is acquitted, or the samples and profiles are no longer necessary for the purpose for which they were supplied.

The Italian Data Protection Authority supervises the DNA database, while the National Committee for Biosafety and Biotechnologies and Life Sciences (CNBBSV) is responsible for monitoring the activities of the central laboratory (Art. 15).

Each year, it is the duty of the Minister of the Interior and the Minister of Justice to inform Parliament on the activities of the DNA database and Central laboratory (Art. 19) (Marchese et al. 2013; Tozzo and Caenazzo 2013).

Different biobanks for research on different diseases are set up in our university/hospital departments. However, current policy approaches to social and ethical issues surrounding biobanks manifest lack of public information given by researchers and government, despite the evidence that Italian citizens are quite optimistic about biotechnologies (Gaskell et al. 2011, Gaskell et al. 2013). There has been increasing interest in conducting public involvment exercises on a large range of issues related to biotechnologies, including many related to biobanks. Knowing the public’s attitudes towards participation in a biobank and biobank management is important and deserves investigation. For this reason, the focus of our survey was to interview our University’s students on these aspects, specifically related to biobanks.

We also collected information about subjects’ attitudes regarding the collection and storage of their own biological samples and related data (DNA profile) for forensic purposes, to evaluate the extent to which young citizens share views and concerns on research and forensic biobanks. In fact, as said before, some people think that there are clear benefits to maintaining a forensic DNA databases holding information from everyone in a country, while others have concerns about privacy, data security, and fairness. Questions about whose DNA profiles should be retained on the DNA database and for what purposes and oversight of national DNA databases continue to be debated in many countries (Machado and Silva 2014; Euroforgen 2017).

In Italy, in 2015, the Italian Government used TV advertising to promote the constitution of the National Forensic DNA Database to combat crime, explaining its importance for the legal system, without promoting any public structured debate.

For all the reasons above reported we conducted the present study, in which a questionnaire was administered to a group of Padua University students to conduct an empirical assessment of individual perceptions and collective attitudes to the risks and benefits of biobanks for medical research and DNA databases for forensic purposes. Our specific aim was to analyze the willingness of this particular category of citizens to donate biological samples to a biobank and/or to be included in the National Forensic DNA Database, and the reasons behind their answers.

## Materials and methods

Once the general aims of the study had been established, a questionnaire was developed by the research team. A homogeneous sample was selected and administered the questionnaire, and a statistical analysis was conducted on the resulting data.

A printed-paper format was preferred for the questionnaire, which was distributed to university attending three different courses: law, medicine and professional nursing. This approach made it easy for respondents to answer the questions and consequently proved an efficient way to obtain a large number of completed questionnaires. At the end of their lectures, students were given a brief explanation of the purpose of our study (in the lecturer’s presence), before the questionnaires were distributed.

The questionnaire comprised five sets of questions covering respondents’:

1. socio-demographic characteristics (gender, age and education);
2. basic knowledge about biobanks;
3. knowledge about Italian legislation on biobanks, and about the National Forensic DNA Database;
4. perception of the benefits and risks of biobanks for research purposes, and willingness to donate their own samples;
5. perception of the benefits and risks of forensic DNA databases, and willingness to have their own DNA profile included in the National Forensic DNA Database.

During one academic semester in 2015, a total of 959 questionnaires were collected, and the data were input in an Excel file for statistical analysis using the Statistical Package for the Social Sciences. We initially used descriptive statistics, classifying and tabulating the data in absolute and relative frequency tables. The *X* ^2^ test was used to analyze respondents’ willingness to have their own biological samples or DNA profile included in the biobank and/or the Italian National Forensic DNA Database, comparing the answers by gender and type of university course.

## Results

The 959 questionnaires (Table 1 contains the questionnaire) that were completed and returned were analyzed by gender and type of university course. The sample of respondents consisted of 639 women (66.6%) and 320 men (33.4%), who ranged in age from 19 to 24 years old.

**Table 1:** English translation of the questionnaire used in the study

Age	
○	17–23
○	24–30
○	31–40
○	41–50
○	50–60
○	≥ 61
Gender	
○	Male
○	Female
University course	
○	Law
○	Medicine
○	Professional nursing
○	PhD
What is a biobank?	
1.	The name of a bank.
2.	A bank for storing biological samples.
3.	A bank for storing biological samples and related data.
4.	I don’t know.
What are the purposes of a biobank?	
1.	Only for research.
2.	Only for legal purposes (to combat crime).
3.	There is only one type, for research and legal purposes.
4.	There are separate biobanks for research and legal purposes.
Are there any research biobanks in Italy?	
1.	YES
2.	NO
3.	I DON’T KNOW
Are there any laws in Italy governing forensic DNA databases?	
1.	YES
2.	NO
3.	I DON’T KNOW
Is there a national forensic DNA database in Italy?	
1.	YES
2.	NO
3.	I DON’T KNOW
Would you be willing to donate your biological sample to a biobank for biomedical research purposes?	
1.	Only if it will be of direct benefit to my health, or if I will be informed about any risks to my health.
2.	No, because it would invade my privacy.
3.	Everyone should donate samples to the biobank to support biomedical research for the common good.
What is the purpose of a forensic DNA database?	
1.	To protect society against crime.
2.	To have a more efficient legal system.
3.	To identify criminals.
4.	To keep criminals under control.
5.	For research.
Would you agree to having your DNA profile stored in a forensic database for legal purposes?	
1.	YES, everyone should be included in the database to help fight crime. If you have nothing to hide, you have nothing to fear.
2.	NO, inclusion on the database is a violation of my individual privacy: I’m not a criminal.
3.	I DON’T KNOW. It depends on how it is regulated, and whether there is sufficient control over access to, and use of the database.

The first multiple-choice question concerned the respondents’ understanding of the meaning of the term “biobank”. The possible answers were:

1. The name of a bank;
2. A bank for storing biological samples.
3. A bank for storing biological samples and related data.
4. I don’t know.

The correct answer (item No. 3) was chosen by 83.7% of the sample.

Concerning the purposes of a biobank, 17.8% answered that it was “Only for research”, 0.7% that it was “Only for legal purposes (to combat crime)”, 46.3% that “There is only one type, for research and purposes”, and 35.1% that “There are separate biobanks for research and legal purposes”. Respondents thus showed a general lack of knowledge regarding the distinction between research and forensic biobanks.

Regarding their awareness of the existence of biobanks in Italy, there were three questions with multiple-choice answers (“Yes”, “No”, or “I don’t know”). As summarized in Table 2, the students tended to answer “I don’t know”: this was true of 52.3% of the students for the first question, 71.3% for the second, and 44.2% for the third.

**Table 2:** Results of three questions regarding the knowledge on the Italian situation

	YES	NO	I DON’T KNOW
Are there any research biobanks in Italy?	*N* = 421	*N* = 36	*N* = 502
	43,9%	3,8%	52,3%
Are there any laws in Italy governing forensic DNA databases?	*N* = 146	*N* = 129	*N* = 684
	15,2%	13,5%	71,3%
Is there a national forensic DNA database in Italy?	*N* = 352	*N* = 183	*N* = 424

As for their willingness to donate their own samples to a biobank for research purposes, the students answered: “Only if it will be of direct benefit to my health, or if I will be informed about any risks to my health” (33.3% of the sample); “No, because it would invade my privacy” (9%); “Everyone should donate samples to the biobank to support biomedical research for the common good” (57.7%).

More females were willing to donate their biological samples than men (34.1% vs 31.6%, *P* = 0.017), and more students of medicine or professional nursing were willing to do so than law students (with 22 and 22.2% versus 13.5%, respectively). The complete results are given in Tables 3 and 4.

**Table 3:** Attitudes towards the willingness to donate to a research biobank, by sex

	YES individualistic position	NO	YES solidaristic perspective	Total	*p*-value
	319 (33,27%)	87 (9,07%)	553 (57,66%)	959 (100%)	
Sex					
Female	218 (34,1%)	46 (7,2%)	375 (58,7%)	639 (66,6%)	0,017
Male	101 (31,6%)	41 (12,8%)	178 (55,6%)	320 (33,4%)	
University Course					
Law	96 (37,5%)	31 (12,1%)	129 (50,4%)	256 (26,7%)	0,001
Medicine	84 (25,8%)	31 (9,5%)	211 (64,7%)	326 (34,0%)	
Professional nursing	139 (36,9%)	25 (6,6%)	213 (56,5%)	377 (39,3%)

**Table 4:** Attitudes towards the willingness to donate to a research biobank, by university course

			Would you be willing to donate your biological sample to a biobank for biomedical research purposes?			
			YES individualistic position	NO	YES solidaristic perspective	Total
University Course	Law	N=	96	31	129	256
		% same course	37,5%	12,1%	50,4%	100,0%
		% Total	10,0%	3,2%	13,5%	26,7%
	Medicine	N=	84	31	211	326
		% same course	25,8%	9,5%	64,7%	100,0%
		% Total	8,8%	3,2%	22,0%	34,0%
	Professional nursing	N=	139	25	213	377
		% same course	36,9%	6,6%	56,5%	100,0%
		% Total	14,5%	2,6%	22,2%	39,3%
Total		N=	319	87	553	959

When respondents were asked about the purpose of a national forensic DNA database, 42.7% chose the answer “To have a more efficient legal system”, 32.5% “To identify criminals”, and 5.2% “To protect society against crime”.

As for the idea of being included in such a database, 35% would agree to having their genetic profile in the National Forensic DNA Database, while nearly one in five (14.5%) would refuse, and the majority (50.5%) might agree under certain conditions and legal provisions. When the responses to these two last questions (Table 5) were combined using the chi-square test (*p* = 0.004), the answer “To protect society against crime” revealed a greater relevance as a reason for agreeing to having one’s DNA profile included in the National Forensic DNA Database.

**Table 5:** Results regarding attitudes regards inclusion in a forensic DNA database under awarness about its scope

			Would you agree to having your DNA profile stored in a forensic database for legal purposes?		
			I DO NOT KNOW	NO	YES
What is the purpose of a forensic DNA database? (*p*-value = 0,004)	1	*N* = 50	20	5	25
		5,2%	40,0%	10,0%	50,0%
	2	*N* = 409	196	50	163
		42,7%	47,9%	12,2%	39,9%
	3	*N* = 312	164	59	89
		32,5%	52,6%	18,9%	28,5%
	4	*N* = 71	40	13	18
		7,4%	56,3%	18,3%	25,4%
	5	*N* = 117	64	12	41
		12,2%	54,7%	10,3%	35,0%
Total		*N* = 959	484	139	336
		100%	50,5%	14,5%	35,0%

1. To protect society against crime. 2. To have a more efficient legal system. 3. To identify criminals. 4. To keep criminals under control. 5. For research

## Discussion

The complexity and ambivalence of public opinion concerning biobanks for research and forensic DNA databases indicate that there is a need to explore the population’s knowledge of this issues more thoroughly, as well as their willingness to donate samples to biobanks in general. The question of public participation is fundamental in the governance of biobanks and has been framed in two different ways, focusing on people providing biological material for research biobanks on the one hand, and on people sharing in the decision-making involved in these projects (Machado and Silva 2015).

Because of their value in criminal investigations, forensic DNA databases are used in a completely different context from biobanks for research purposes, and the former are controlled by state authorities or police services under specific legal provisions. A society wishing to make use of a system of forensic DNA databases has to be prepared to make the fundamental choice between security and personal freedom. People’s opinions on forensic DNA databases are consequently a tangible sign of how they judge the efficiency of their country’s legal system, and how much trust they place in the government. As reported by Zadok et al., improving public trust in the governance and use of forensic DNA databases may result in making citizens more likely to agree to give up their right to privacy to some extent with the aim to protect the common good and to assure that privacy right and the security of society will not conflict but instead complement each other (Zadok et al., 2010).

This study investigated what a sample of students at Padua University (Italy) knew and thought about biobanking and DNA profiling, taking a bottom-up approach to examining this particular population’s willingness to donate biological samples to a biobank for research purposes and/or have their DNA profile included in the National Forensic DNA Database, and the reasons behind their answers. The vast majority of the students invited to take part in the survey answered the questionnaire, making up a representative sample of the target population.

Although our questionnaire was administered to a particular sample of well-educated young Italian adults, which cannot represent the Italian population at large, our findings suggest a generally positive attitude to the idea of donating biological samples to a biobank for research purposes, confirming a previous report from Porteri et al. (2014) in a survey conducted in a smaller, different sample population.

While a relatively large number of respondents seemed to be familiar with the term ‘biobank’, many had no clear idea of the distinction between biobanking (for medical research purposes) and forensic DNA profiling: numerous respondents chose “I don’t know” for the questions specifically focusing on Italy’s legislation concerning the latter (Table 2). This is despite the attention paid by the media in the weeks and months before the students answered our questionnaire, when the Italian Government used TV advertising to promote the constitution of the National Forensic DNA Database to combat crime, explaining its importance for the legal system.

When we examined how willing respondents would be to donate samples to a biobank, 33.3% of the sample would do so only for personal gain (“Only if it will be of direct benefit to my health, or if I will be informed about any risks to my health”), while 57.7% would do so for altruistic reasons (“Everyone should donate samples to the biobank to support biomedical research for the common good”). Females were reportedly more willing to do so than males (34.1 vs 31.6%) and, although such a small difference between the two genders makes it impossible to draw any conclusions as yet, it would be interesting to delve more deeply into people’s different attitudes from a gender perspective (Lawrence and Anita 2007; Klinge 2008; Ballantyne and Rogers 2010).

Medical students and professional nursing students (accounting for 22 and 22.2% of the total) seemed to be more willing to donate to a biobank than law students (13.5% of the total). The former’s greater willingness to donate samples probably reflects a more positive attitude to biomedical research, and also supports the conviction that trust crucially influences a person’s decision on these matters (De Vries et al. 2016). In other words, our findings indicate that Italian medical and professional nursing students have more faith and interest in scientific and medical research, whereas law students may be more reluctant to donate their biological samples because they are unfamiliar with, or distrustful of the research institutions and methodologies involved.

Leaving aside their motives, our respondents showed a broad willingness to contribute to biobanking schemes, with 91% of the students reportedly prepared to donate their samples for research purposes, as previously reported in another study on Italian people’s attitude to this issue (Porteri et al. 2014).

Previous research on public attitudes and opinions concerning forensic DNA databases identified shared concerns and homogeneous perceptions on several aspects, revealing a common body of knowledge on the ethical, legal and social issues related to DNA databases (Gamero et al. 2008; Machado and Silva 2014).

In contrast, our findings point to a shortage of knowledge in our sample regarding such databases, and particularly their forensic applications.

Although our respondents tended to be unaware of the existence of a law concerning the National Forensic DNA Database, 35% of them were reportedly willing to have their DNA profile included in this database. They seemed to have little idea of the benefits and risks of such action, and their reasons for agreeing or refusing to have their genetic profile stored in the database.

In particular, we want to underline that the Italian Forensic DNA database has yet to come into effect, so the correct answer to our question “Is there a national forensic DNA database in Italy?” was “No”, but it was only chosen by 19.1% of our sample.

The sample’s tendency to accept the idea of a national database seems to reflect an unexpectedly high degree of trust placed by our university students in the ability of the state to manage such a system (Italian citizens generally have less confidence in their country’s judiciary than in most other European countries). The percentage of our respondents willing to have their genetic profile included in the National Forensic DNA Database was higher among those who saw such a database as a powerful tool for improving the efficiency of the criminal justice system, and this probably reflects the conviction that “If you have nothing to hide, you have nothing to fear”.

## Conclusion

Although our questionnaire was administered to a sample scarcely representative of the Italian population at large, our findings shed some light on the general attitude of young, well-educated Italian people to the donation of biological samples for use in medical research and forensics.

While there are limitations of this study referring to the choice of a particular category of University students, our results contribute to an empirically-grounded understanding of what citizens know about biobanking and DNA profiling, and their willingness to donate samples to a biobank for use in medical research and/or to have their genetic profile included in a national forensic DNA database. Our findings may have clear implications for the policy discussion on biobanks in Italy. With reference to this aspect, it seems more than ever appropriate to implement the system of involvement, education and information to the population about the aims of research biobanks, in the light of what seems to be a positive attitude of Italian younger citizens to the donation of their biological samples for research purposes, which should be recognized and further motivated and implemented, in order to improve the public trust in research biobanks.

Moreover, it is appropriate to take into account the actual level of Italian population’s awareness of DNA database applications for forensic purposes, which seems to be poor, in order to ensure a better interaction between policy makers and citizens and to make them more aware of the need to balance the security of society with the rights of the individual.

The complexity and ambivalence of the public’s understanding and views on these issues point to a need for more research to explore people’s beliefs about the risks and benefits of these technologies in finer detail. This would facilitate the promotion of public participation in biobanking schemes, and enable the potential of biobanks to be fully exploited in a framework in which public attitudes are respected.
